# Expectation as a Risk for Covid‐19‐Related Olfactory Changes: Observations From the California Farmworkers Health Survey

**DOI:** 10.1111/risa.70177

**Published:** 2026-01-30

**Authors:** Derry Ridgway, Nimrat K. Sandhu, Ana M. Mora, Katherine Kogut, Paul Brown, Brenda Eskenazi

**Affiliations:** ^1^ Health Science Research Institute University of California Merced California USA; ^2^ Department of Public Health University of California Merced California USA; ^3^ Center for Environmental Research and Community Health School of Public Health University of California Berkeley California USA

**Keywords:** Covid‐19, Rubin Table, smell, taste

## Abstract

Some outcomes in medical and public health research, as well as clinical practice, must rely on patient reports and may be influenced by the prior knowledge of the patient. During the early months of the SARS‐CoV‐2 epidemic, changes in the sense of smell and taste were widely reported as a distinctive aspect of the new respiratory contagion. Using a Rubin Model of causal inference and data from a California Department of Public Health–sponsored survey of California farmworker health, we estimate that approximately half (56.5%) of infected patients reporting olfactory changes after a diagnosis of Covid‐19 would not have reported olfactory changes if not made aware of their Covid‐19 infection. The observations support a similar conclusion with respect to Covid‐19‐related changes in the sense of taste.

## 1 Introduction

During the early months of the Covid‐19 epidemic, the symptomatology and clinical course of infections with SARS‐CoV‐2 were extensively publicized and discussed. Changes in the sense of smell, and to a lesser extent changes in the sense of taste, were identified as more prominent for Covid‐19 victims compared to patients with other familiar respiratory viral diseases. Prior to the availability of nucleic acid and then antigen tests for SARS‐CoV‐2, loss of olfaction or taste was understood by many to allow differentiation between Covid‐19 and other respiratory infections (Giacomelli et al. 2020; Marshall 2022).

Recent research relates the pathophysiology of the olfactory symptoms to downregulation of the signaling function of nasal receptors without direct infection of neurons. Identified risk factors associated with the frequency, duration, and severity of olfactory dysfunction include the infecting SARS‐CoV‐2 variant, the time of infection during the epidemic, and certain genetic characteristics of the patients (Marshall 2022; Cattaneo et al. 2022). Exposure to one of the Covid‐19 vaccines might also affect the risk for olfactory dysfunction. In most clinical settings, assessments of olfactory and gustatory functions are limited to patient report. It is possible that for these well‐publicized and subjectively assessed symptoms of Covid‐19, a patient's prior knowledge and expectation might affect the likelihood of experiencing and reporting the symptoms.

When controlled investigations such as randomized trials are unethical or impractical, we may be limited in our ability to assess the causal contribution of alternative events. In our case, we cannot know for certain whether SARS‐CoV‐2 infection would cause olfactory or taste loss in a particular patient, both with and without prior expectation. Paul Holland has termed this “the fundamental problem of causal inference” (Holland 1986).

We apply a statistical methodology described by Don Rubin and Paul Holland (Rubin 1974) to answer the question: Among patients who report smell (or taste) changes after infection diagnosed as Covid‐19, how many would not have reported olfactory (or taste) loss if they had not been aware of their infection?

The Rubin analysis applies a single step of inferential reasoning to an observational dataset to allow derivation of a numerical estimate for a counterfactual question such as the one proposed above.

## 2 Methods

As part of a large survey of the health status of California farmworkers conducted during the late winter and spring of 2022 (2022 California Farmworkers Health Survey; CFHS), we asked 297 subjects about their experience with Covid‐19 and recent changes in their sense of smell and taste^1^. In addition, we performed serologic testing for anti‐nucleocapsid (anti‐N) IgG antibodies to SARS‐CoV‐2^2^ and administered an 8‐item test of olfaction (NHANES Pocket Smell Test; On, Moein, Khan, and Doty 2025). Mora et al. (2023) and Brown, Flores, and Padilla (2022) provide detailed descriptions of the study methods, including subject recruitment and data management (Mora et al. 2023; Brown, Flores, and Padilla 2022).

## 3 Results

### 3.1 Primary Data

#### 3.1.1 Subject Demographics

All subjects were adults who were employed as agricultural workers in California (field work, packing house, nursery, or other) during the previous 12 months. The study population is briefly described in Table 1. Mora et al. (2023) and Brown et al. (2022) provide additional demographic details (Mora et al. 2023; Brown et al. 2022).

**TABLE 1:** Study population.

Subject demographic characteristics	Number (% of 297)
Female	176 (59%)
Born in Mexico or Central America	268 (90%)
Language spoken at home is Spanish	273 (92%)
Years in the United States, ≤ 20	115 (39%)
Primary work activity in the field	238 (80%)

#### 3.1.2 Covid‐19 Infection

As shown in Table 2, at the time of our survey, 20+ months into the Covid‐19 epidemic, 193 of our 297 subjects (65%) had experienced SARS‐CoV‐2 infection, based on either positive history of illness or anti‐SARS‐CoV‐2 (N) positive test or both.

**TABLE 2:** Summary of Infection with SARS‐CoV‐2.

Subject reported history of infection	All subjects, *n* (% of all subjects)	Anti‐SARS‐CoV‐2 nucleocapsid antibody positive, *n* (% of subjects in Covid category)	Anti‐SARS‐CoV‐2 nucleocapsid antibody negative, *n* (% of subjects in Covid category)
No known or suspected infection	145 (49%)	41 (28%)	104 (72%)
Suspected infection without test confirmation^a^	28 (9%)	14 (50%)	14 (50%)
Test‐confirmed infection^b^	124 (42%)	64 (52%)	59 (48%)

^a^ The reason a subject might have experienced a suspected episode of Covid‐19 without a confirmatory test was not recorded. During the early months of the epidemic, Covid‐19 diagnoses were commonly made without confirmatory testing because no test was available or because the scarcity of tests and clinical status of the patient combined to make testing unadvised.

^b^ One subject had missing anti‐SARS‐CoV‐2 nucleocapsid antibody test.

Of importance for our purposes, 41 of 145 subjects with no reported history of Covid‐19 infection had positive antibody tests confirming prior infection. We term the cohort of 152 subjects with suspected or test‐confirmed Covid‐19, “Overt Covid,” and the cohort of 41 subjects with positive antibody tests but no history of infection, “Covert Covid.”

#### 3.1.3 Smell and Taste

Subject reports of changes in their sense of smell or taste are summarized by category of Covid‐19 infection in Table 3.

**TABLE 3:** Summary of subject‐reported changes in smell and taste.

Covid‐19 infection status	All subjects, *n* (% of all subjects)	Perceived smell problems, *n* (% of subjects in Covid category)	Perceived taste problems, *n* (% of subjects in Covid category)
No Covid^a^	104 (35%)	14 (13%)	5 (5%)
Overt Covid^b^	152 (51%)	51 (34%)	37 (24%)
Covert Covid^c^	41 (14%)	6 (15%)	1 (2%)

^a^ No Covid = no historic or serologic evidence of infection.

^b^ Overt Covid = any subject reporting a history of suspected and/or test‐confirmed Covid‐19, whether or not anti‐SARS‐CoV‐2 nucleocapsid antibody was positive.

^c^ Covert Covid = any subject reporting no history of Covid‐19 and with positive anti‐SARS‐CoV‐2 nucleocapsid antibody test.

Some subjects with Overt Covid (subjects aware of having experienced infection with Covid‐19; *n* = 39) were able to rate the severity of their illness from 1 = very mild to 10 = extremely serious (initial illness for subjects with more than one Covid‐19 episode). The mean severity ratings for subjects with and without reported smell or taste problems were 5.78 compared to 5.96 (smell problems; no smell problems) and 7.0 compared to 5.9 (taste problems; no taste problems).

On the 8‐item NHANES test of olfaction, scores of 7 or 8 are judged normal [8] [On 2025]. The number (%) of subjects with scores of 7 or 8 were: No Covid, 73/104 (70%); Overt Covid, 116/151 (77%); and Covert Covid, 27/41 (66%).

### 3.2 Causal Analysis of the Effect of Subject Awareness of Covid Infection

We can craft Rubin Causal Model Tables using our survey data for subjects with reported changes in sense of smell and taste. For changes in the sense of smell, normalized observed results populate the marginal totals, labelled A, B, C, and D, as shown in Table 4.

**TABLE 4:** Observed subject‐reported changes in smell following covid infection (Cells A, B, C, and D) and derived subject condition (Cells E, F, G, and H).

	Patients with smell problems if not aware of Covid infection (Covert Covid)	Patients with no smell problems if not aware of Covid infection (Covert Covid)	Total (patients per 1000)
Patients with smell problems if aware of Covid infection (Overt Covid)	E	F	A = 336
Patients with no smell problems if aware of Covid infection (Overt Covid)	G	H	B = 664
Total (patients per 1000)	D = 146	C = 854	1000

We can make the reasonable assumption that there are no subjects who reported smell problems after Covert Covid who would have reported no smell symptoms if they had been aware of their Covid infection. Thus, G in Table 4 equals 0. From that assumption and the observation‐based margin totals, we derive E = 146, H = 664, and F = 190. Cell F represents an estimate of subjects with smell problems if and only if aware of Covid infection. Based on our early 2022 observations of California farmworkers, of 1000 patients diagnosed with Covid‐19, 336 will report olfactory changes. Of those 336, 190 (56.5%) would not have reported olfactory changes if they had not been aware of their SARS‐CoV‐2 infection.

Using normalized CFHS observations related to taste and a similar assumption for Cell G, we can craft a Rubin Table for changes in taste following Covid infection, Table 5. Based on our early 2022 observations of California farmworkers, of 1000 patients diagnosed with Covid‐19, 245 will report changes in taste. Again, we complete the table by reasoning that there are no subjects who reported taste changes after Covert Covid who would have reported no taste symptoms if they had been aware of their Covid infection (G = 0). Of the 245 patients reporting taste changes, 221 (90.2%) would not have reported taste changes if they had not been aware of their SARS‐CoV‐2 infection.

**TABLE 5:** Observed subject‐reported changes in taste following covid infection (Cells A, B, C, and D) and derived subject condition (Cells E, F, G, and H).

	Patients with taste problems if not aware of Covid infection (Covert Covid)	Patients with no taste problems if not aware of Covid infection (Covert Covid)	Total (patients per 1000)
Patients with taste problems if aware of Covid infection (Overt Covid)	E = 24	F = 221	A = 245
Patients with no taste problems if aware of Covid infection (Overt Covid)	G = 0	H = 755	B = 755
Total (patients per 1000)	D = 24	C = 976	1000

Conclusions: Approximately half of patients diagnosed with Covid‐19 who report olfactory changes after their illness would not report olfactory changes if they had not been aware of the infection; similarly, approximately 90% of patients diagnosed with Covid‐19 who report changes in taste after their illness would not report taste changes if they had not been aware of the infection.

## 4 Discussion

### 4.1 Clinical Implications

An undesired medical effect that exhibits increased frequency and/or severity because of patient anticipation is a well‐described phenomenon. With reference to the adverse side effects of pharmaceuticals, it is called the nocebo effect (Nasiri‐Dehsorkhi, Vaziri, Esmaillzadeh, and Adibi 2024). Our result suggests a similar association between patient expectation and the reports of olfactory or gustatory changes after infection with SARS‐CoV‐2. With respect to a planned intervention, clinicians wrestle with the ethical implications of explaining potential side effects and the associated risk that the likelihood or severity of the side effect is increased by the patient's knowledge, versus paternalistic limiting of explanations about potential adversities.

Our Rubin analysis has shown a contribution of patient expectation on the frequency of reports of smell and taste effects after Covid‐19. While there is no current well‐described intervention to correct Covid‐19‐related loss of smell or taste, the observed nocebo effect may suggest that even an intervention with limited pharmacodynamic effect might be successful for many patients.

For investigators, however, the lesson may be cautionary. Any controlled efficacy trial of a corrective intervention could encounter a very strong placebo effect, requiring large patient numbers to achieve statistical separation.

We propose that the difference in olfactory and taste changes between subjects with overt and covert Covid‐19 infection is related to prior knowledge—expectation—of these effects, similar to the nocebo effects observed for clinical interventions. The FWHS did not include any formal evaluation of patients’ awareness of olfactory or taste effects of Covid‐19 infection. Our attribution depends on the assumption that our California farmworker subjects were alert to the well‐publicized aspect of the new respiratory disease; no alternative explanation suggests itself.

### 4.2 Causal Inference

The logic of causality and the mathematics of statistical inference are distinct enterprises (Pearl 2010). When an observed dataset allows Rubin/Holland reasoning, we have the opportunity to make numerical estimates for counterfactual questions with very limited knowledge of the underlying probabilistic distributions (Rosenbaum 1984). We were able to take advantage of just such a circumstance with our retrospective Covid‐19 data among California farmworkers. Additional points deserve mention.

First, our results concern symptoms, changes in the senses of smell and taste, that commonly rely on subjective reporting. In our study, the objective 8‐item NHANES Pocket Smell Test showed little difference among our Covid‐defined cohorts; no formal follow‐up testing was undertaken for subjects with abnormal scores (scores <7). The application of Rubin/Holland reasoning is unrelated to the objectivity or subjectivity of the clinical findings. For an example of Rubin analyses applied to strictly objective medical outcomes, see Ridgway (2000), addressing the question of paralysis following alternative methods of vaccination against poliomyelitis (Ridgway 2000). Further, our findings do not suggest that the changes in smell and taste were “unreal” or present only in the imaginations of our subjects. There is now extensive literature with objective documentation of sensory losses following SARS‐CoV‐2 infection; see, for example, Sharetts et al. (2024).

Second, in our Rubin analyses, we were able to reason that one cell of our Rubin Table would be empty: no patient who had loss of smell or taste after Covert Covid would have reported no such loss if aware of the infection. Explanations for a patient who reported loss of olfactory or taste sense without knowing about his infection and would fail to report the sensory change when aware of having Covid‐19 might include deliberate contrariness or misdirection from a clinician. These possible explanations seem very unlikely in the setting of our CFHS. While the assumption of a zero‐value for one cell makes the melding of arithmetic and reasoning easy, a zero‐value is not a Rubin/Holland Table requirement. We need only find an external justification for entering a value in one or more of the open cells. For example, we might imagine an intervention that would protect the signaling capacity of nasal receptors if applied early in the illness with, say, 50% efficacy. If available to our subjects, then among patients who would experience loss of smell if unaware of infection (Table 3, Cell D = 146), half would and half would not experience loss of smell if aware of their infection (Cell E = 73, Cell G = 73), because of the timely application of our imagined intervention. These assumed hypothetical non‐zero values would allow us to complete the table.

### 4.3 Strengths and Limitations

The CFHS population, including the subpopulation reported in this study, examined a sample of convenience and may not represent the overall farmworker population. The design of the planned CFHS was modified in 2020 when the unexpected Covid‐19 epidemic became an important aspect of California farmworkers’ health experience. The study took advantage of the planned CFHS support structure to add assessments of the health implications of the new contagion. We did not anticipate the imbalanced sensory observations in patients with overt and covert infections. Fortunately, the causal inferential analyses developed by Don Rubin and Paul Holland are well suited to retrospective findings such as ours (Holland 1986; Rubin 1974; Rosenbaum 1984).

The precision of our estimates is limited by the small number of observations in each group, especially among subjects with Covert Covid. Our data are counts and cannot support variance‐ or percentile‐based sensitivity calculations. The count data for sensory loss among Covert Covid subjects are linearly related to our final result, allowing a One‐at‐a‐Time (OAT) sensitivity analysis. Each Covert Covid subject with loss of smell accounts for approximately 7% of our final estimate; using an OAT sensitivity calculation, a single additional Covert Covid subject who reported change in the sense of smell would change our estimate for the number of patients who would not experience change if not aware of Covid‐19 infection from 56.5% to 49.1%. Using a similar OAT analysis, each unit change in the number of Overt Covid patients with loss of smell would result in a local change in the final result of less than 1% (calculations not shown).

It is possible that the severity of Covid‐19 is associated with the frequency of olfactory and taste changes; to date that relationship is not well defined (Sharetts et al. 2024; Vaira et al. 2020). We can reason, without confirmatory data, that subjects with Covert Covid experienced, on average, less severe disease. As noted above, among our cohort of patients with known Covid‐19 infection, the severity of illness was not associated with the likelihood of reporting changes in taste or smell.

Our subjects were California farmworkers; > 90% reported Latino ethnicity with Spanish or Indigenous as their primary language. Authors studying other conditions, such as heat‐ or pesticide‐related illness, have reported that similar populations have limited health literacy (Smith, Ferranti, Hertzberg, and Mac 2021). Observations about our subjects’ medical knowledge and symptoms may generalize poorly to other populations. We have no reason to expect that the nocebo‐like effect seen in our farmworkers would not be found in other patient groups.

## Author Contributions

All authors contributed to the design and conduct of the 2022 California Farmworkers Health Survey and the sub‐study conducted in Monterey and Tulare Counties. DR prepared the manuscript and is the corresponding author.

## Conflicts of Interest

The authors declare no conflicts of interest.

## Data Availability

The data that support the findings of this study are available from the authors upon reasonable request.

## Endnotes

1 Subjects were asked, ‘During the last month, have you had a problem with your ability to smell, such as not being able to smell things or things not smelling the way they are supposed to?‘ There was a similar question about taste.

2 The anti‐SARS‐CoV‐2 nucleocapsid antibody test detects circulating antibodies after SARS‐CoV‐2 infection but not after exposure to any of the available vaccinations without infection.
